# Association of consumption of sugar-sweetened beverages with cognitive function among the adolescents aged 12–16 years in US, NHANES III, 1988–1994

**DOI:** 10.3389/fnut.2022.939820

**Published:** 2022-08-11

**Authors:** Xiaofang Yan, Yingxia Xu, Jitian Huang, Yanmei Li, Qian Li, Juan Zheng, Qingsong Chen, Wenhan Yang

**Affiliations:** ^1^Department of Child and Adolescent Health, School of Public Health, Guangdong Pharmaceutical University, Guangzhou, China; ^2^Baiyun Maternity and Child Healthcare Hospital of Guangzhou, Guangzhou, China; ^3^Department of Occupational Health, School of Public Health, Guangdong Pharmaceutical University, Guangzhou, China

**Keywords:** sugar-sweetened beverages, cognition, adolescent, NHANES III, WISC-R, WRAT-R

## Abstract

**Objective:**

As a major source of added sugar, the consumption of sugar-sweetened beverages (SSBs) continues to increase worldwide. The adverse health effects associated with SSBs are also risk factors for cognitive development, but studies on the relationship between SSBs and adolescents' cognitive function are limited. We used data released by the National Health and Nutrition Examination Survey (NHANES) III (1988–1994) to explore the association between the consumption of SSBs and cognitive function among children and adolescents aged 12–16 years in the United States.

**Methods and procedures:**

A nationally representative population sample included 1,809 adolescents aged 12–16 years who participated in the United States NHANES from 1988 to 1994 and provided samples for the dietary intake frequency questionnaire and measures of cognitive function. Binary logistic regression was used to estimate the association between the frequency of SSB consumption and scores on cognitive function tests.

**Results:**

This study of 1,809 adolescents aged 12–16 years comprised 963 girls (weighted proportion, 48.17%) and 846 boys (weighted, 51.83%), with a weighted mean (SE) age of 13.99 (0.05) years. Compared with adolescents who intake SSBs 0–1 times per week, those who drank 4–7 times per week had better scores in arithmetic, reading, and digit span tests, with odds ratios (ORs) of 0.36 (95% CI = 0.16–0.82), 0.35 (95% CI = 0.18–0.70), and 0.19 (95% CI = 0.08–0.44), respectively. The ORs for abnormal block design scores increase with the frequency of SSB intake after being adjusted for potential confounders (*P* for trend 0.02). Stratified analyses showed that compared with normal or below BMI, among overweight or obese individuals, the frequency of SSB intake had significant ORs for abnormal digit span scores (OR = 4.76, 95% CI = 1.19–18.96 vs. 0.35, 95% CI = 0.10–1.25; *P* for interaction = 0.01).

**Conclusion:**

The positive associations of SSBs at moderate level intake with better scores in arithmetic, reading, and digit span were observed, but no dose-response relationship was identified at the overall level. Additionally, with the increasing frequency of SSB consumption, the risk of anomalous block design scores increased among US adolescents. Further investigation is warranted to confirm the association and mechanism between SSBs and cognitive function among adolescents.

## Introduction

Sugar-sweetened beverages (SSBs) are the major source of added sugar, representing a nutritionally poor, calorie-dense but palatable type of drink that is appealing to children and adolescents (1). The consumption of SSBs continues to increase worldwide. Similarly, American children and adolescents of all ages are increasingly consuming SSBs (2), and the proportion of calories consumed from SSBs also increased significantly (3).

Current studies indicated that increased consumption of SSBs is associated with a number of health risks, including cardiometabolic burden, increased risk of obesity, type 2 diabetes, hypertension, and metabolic syndrome among people of all age groups (4–7). Moreover, regarding its impact on brain function, findings from previous studies suggested that SSBs are associated with the responsive release of dopamine and opioids in the striatum (8), which is related to value-based learning, encoding hedonic valuation, and motivated behavior (9, 10).

During adolescence, the brain is undergoing rapid structural and functional development, and the neurofunction of adolescents can easily be disturbed by external factors during the special period (11, 12). Although the evidence is limited, there are studies relating SSB consumption to mental health problems (13, 14), dysfunction of executive ability (15), and behavioral adaptation (16) in adolescents. Nevertheless, very few studies have focused on the impact of SSB consumption on cognitive function, and the findings remain inconsistent and insufficient, especially in adolescents.

The cognitive function plays an essential role in learning, daily life, and academic performance in early life, which is affected by multifactor including diet (17). It has been widely recognized that excessive sugar or simple carbohydrate intake is linked with impaired cognitive functions (18). A study on adolescent rats showed that excessive consumption of added sugars, especially HFCS-55 (high fructose corn syrup-55), during adolescence, adversely affects hippocampal function and metabolic outcomes and promotes neuroinflammation (19). Regular consumption of sweets or sweeteners, even at low dosages, can significantly alter brain neurochemistry, especially dopamine levels and its turnover rate, as well as high cognitive function (20). A recent cross-sectional study in China showed that SSB consumption was positively associated with all subscales and composite scores of the Behavior Rating Inventory of Executive Function (BRIEF, for assessment of executive function impairment) and a higher risk of increased executive difficulty (21). Another study reported that increased consumption of SSBs was associated with significantly lower test scores in reading and numeracy in Australian school-aged children (22). Therefore, it is reasonable to hypothesize that SSBs may negatively impact cognitive function beyond a certain level of intake.

However, an early meta-analysis from the 90s concluded that sugar did not affect children's behavioral or cognitive performance (23). In addition to that, a double-blinded controlled trial showed that neither sucrose nor aspartame had significant cognitive or behavioral effects on normal preschoolers or school-age children who were considered sugar-sensitive (24). We aimed to address the inconsistency over the potential impact of SSBs on the cognitive function of adolescents.

Therefore, in this study, we used data published by NHANES III (1988–1994) to examine the association between the consumption of SSBs and cognitive function among adolescents aged 12–16 years in the United States.

## Methods

### Study population

Our analysis was based on 1988–1994 cross-sectional data from the NHANES III database, a nationally representative survey regularly conducted by the US Centers for Disease Control and Prevention. A stratified multistage clustered probability design involving two 3-year phases was used to select a sample of the civilian noninstitutionalized US population at and above 2 months of age. Detailed descriptions for the survey are available elsewhere (25).

NHANES III included medical and cognitive examinations and interviews conducted with survey children and proxy respondents. The primary purpose of this study was to examine the relationship between SSB intake and cognitive function in adolescents. In the original study, cognitive examinations were administered to children and adolescents aged 6–16 years, and dietary intake frequency questionnaires were administered to adolescents aged 12–16 years, therefore, restricted our study population to those aged 12–16 years and had cognitive function test results and SSB consumption data (*N* = 1,825). After excluding 9 intellectually disabled and 7 children and adolescents who attended or needed special schools due to health conditions, 1,809 adolescents remained for the primary analysis.

### Measurements and variable definitions

#### Cognitive function assessment

Cognitive function was evaluated using subcomponents of two tests, namely, the Wechsler Intelligence Scale for Children-Revised (WISC-R) and the Wide Range Achievement Test-Revised (WRAT-R). The Mobile Examination Center (MEC) interviewers were trained to conduct the WISC/WRAT examination. During annual site visits, test administration was evaluated, and retraining was performed where necessary. Two subcomponents of the WISC-R test, namely, a verbal component (Digit Span) and a performance exam (Block Design), were administered and are considered relatively culturally unbiased. In addition, two subcomponents of the WRAT-R test, namely, math and reading, were conducted. The WISC-R test was administered first and was followed by the WRAT-R. The scores for all four subcomponents used a common scale and were derived for each child relative to his/her age group based on test-specific standardization samples created by the test developers. This study used scaled scores, which were determined using calculations provided in the WISC-R Manual and WRAT-R Administration Manual (26, 27). The scaled score for the four tests allows comparison between the WRAT-R and WISC-R exams (25). Since the data distribution type is non-normal, bounded by 5^th^ percentile scores, adolescents whose scores are lower than 5^th^ percentile scores and higher than or equal to 5^th^ percentile scores are divided into abnormal group and normal group, respectively (28).

#### Consumption of sugar-sweetened beverages

During the home interview, intakes of SSBs were determined from responses to the food-frequency questionnaire that was administered to participants to assess their usual consumption over the prior month (25). Food-frequency questionnaire assessment of dietary intake has been shown to be a valid and reliable method for assessing average dietary consumption (29–31). Flavored drink such as ginger ale and tonic water was considered SSBs, and mixed drink containing SSBs was also counted for, but carbonated drink without sugar (e.g., club soda or Seltzer) was not included (32). The frequency of SSB intake was converted according to 1 month = 4.3 weeks (values were unrounded) (25), we combined the quartile situation of the data distribution (3.8, 12.7, and 29.2 times/month are approximately equal to 1, 4, and 7 times/week) and the classification method of the other study (33), and the SSB classification for this study also used 1 and 4 as cutoff points. Finally, we selected three nodes (1, 4, and 7 times/week) as the dividing points to divide the SSBs into four groups with average weekly consumption frequency being: [0, 1), [1, 4), [4, 7), and [7, 182) times/week.

#### Assessment of covariates

Information about participant age, gender, race/ethnicity, family income, marital status and educational level of adult reference person [defined as one of the persons in the household who owned or rented the home (most often the parent)], physical activity, and dietary intake was collected using questionnaires. Race/ethnicity was categorized as non-Hispanic white, non-Hispanic black, Mexican-American, and other races/ethnicity. Family income was classified as the ratio of family income to the federal poverty level (≤ 1.30, 1.31–3.50, and > 3.50) (34). The education level of the family reference person was grouped as below high school, high school, and college or above. The marital status of the family reference person was grouped as married/living and as married or not. The adolescents were asked “How many times per week do you play or exercise enough to make you sweat or breathe hard?.” According to the Surgeon General's Report on Physical Activity and Health (35), if adolescents reported participating in physical activity at least five times per week, these adolescents were classified as being physically active most days of the week. Based on the studies linking obesity to cognitive function (36), we examined whether the relationship between frequency of SSB consumption and cognitive function differed by sex, race/ethnicity, and BMI. BMI was calculated in kilogram per meter square and then converted to sex- and age-specific BMI percentile values using a computerized formula derived from the 2,000 Centers for Disease Control Growth Charts (37). We assigned each participant to an obesity BMI stratum (≥95^th^ percentile), an overweight BMI stratum (85^th^ to 94^th^ percentile), or a normal BMI stratum (<85^th^ percentile). The grams of total carbohydrates include sugars and complex carbohydrates. The total carbohydrate figure is the difference between 100 and the sum of the protein, fat, ash, and water (25). According to the 2015–2020 Dietary Guidelines for Americans, the recommended carbohydrate intake for adolescents aged 12–16 years is 45%-65% kcal (38). Therefore, carbohydrate intake was categorized into three groups (< 45%, 45–65%, and > 65% kcal).

### Statistical methods

Following the NHANES III analytic guidelines (39), we applied sampling weights, strata, and primary sampling units in the analyses to account for the unequal probability of selection, oversampling of certain subpopulations, and non-response adjustment.

Means and proportions of baseline characteristics were compared using ANOVA for continuous variables and chi-square tests for categorical variables. We used binary logistic regression to estimate the association between the frequency of SSB consumption and four cognitive functions. Model 1 adjusted for adolescent sex and age, whereas model 2 additionally adjusted for sociodemographic and lifestyle characteristics. In a fully adjusted model, we adjusted for age, sex, race/ethnicity, education level and marital status of family reference person, household income level, physical activity, BMI, and carbohydrate intake. Furthermore, we performed a stratified analysis to examine whether this association differed by sex, ethnicity, and BMI.

All statistical analyses were conducted using the survey modules of SAS software, version 9.4 (SAS Institute). A 2-sided *P*-value < 0.05 was considered statistically significant.

## Results

The characteristics of participants according to their frequency of SSB consumption are described in Table 1. The study population of 1,809 adolescents aged 12–16 years comprised 963 girls (weighted proportion, 48.17%) and 846 boys (weighted, 51.83%), with a weighted mean (SE) age of 13.99 (0.05) years; 486 participants (weighted, 67.74%) of non-Hispanic white, 636 (weighted, 14.48%) of non-Hispanic black, 601 (weighted, 7.92%) of Mexican-American, and 86 (weighted, 9.86%) of other race/ethnicity. Weighted mean (SE) scores for arithmetic, reading, block design, and digit span were 8.54 (0.17), 8.69 (0.16), 9.32 (0.12), and 8.56 (0.11), respectively (not shown in table). There were no differences between the four frequency classes of SSB consumption among those cognitive functions (arithmetic, reading, and block design) in regard to normal and abnormal scores. But, there was a difference in four levels of SSB intake among adolescents with normal and abnormal digit span scores (Table 1).

**Table 1:** Mean (continuous variables) and proportion (categorical variables) differences in cognitive function, socioeconomic status, and demographics of adolescents aged 12–16 years are presented by consumption of sugar-sweetened beverages (SSBs) in NANESIII, 1988–1994.

		**Frequency of intake of SSBs**					
**Characteristics^a^**	**Total**	**[0, 1) times/week**	**[1, 4) times/week**	**[4, 7)** **times/week**	**[7, 182]** **times/week**	***χ^2^/F***	***P*-value**
No. of participants (%)	1,809 (100)	492 (27.20)	669 (35.83)	419 (24.04)	229 (12.93)		
Age, y [mean (SE)]	13.99 (0.05)	13.84 (0.09)	13.89 (0.07)	14.03 (0.06)	14.50 (0.13)	13.88	<0.001
**Sex**, ***N*** **(%)**							
Male	846 (51.83)	198 (41.26)	332 (54.70)	211 (59.28)	105 (52.29)	13.66	0.01
Female	963 (48.17)	294 (58.74)	337 (45.30)	208 (40.70)	124 (47.71)		
**Race/ethnicity**, ***N*** **(%)**							
Non-Hispanic white	486 (67.74)	120 (62.86)	173 (65.73)	126 (71.65)	67 (76.27)	22.88	0.01
Non-Hispanic black	636 (14.48)	194 (16.16)	214 (14.01)	140 (12.86)	88 (15.28)		
Mexican-American	601 (7.92)	149 (7.93)	250 (9.23)	132 (6.87)	70 (6.22)		
Other	86 (9.86)	29 (13.06)	32 (11.02)	21 (8.62)	4 (2.23)		
**Family reference person married/living as married**, ***N*** **(%)**							
Yes	1,242 (74.81)	350 (75.77)	463 (73.55)	284 (77.27)	145 (71.74)	1.22	0.75
No	567 (25.19)	142 (24.23)	206 (26.45)	135 (22.73)	84 (28.26)		
**Family reference person education level**, ***N*** **(%)**							
Below high school	510 (15.84)	124 (15.52)	198 (12.63)	113 (17.94)	75 (21.50)	6.24	0.38
High school	815 (43.56)	229 (42.32)	293 (43.55)	193 (44.34)	100 (44.72)		
College or above	484 (40.61)	139 (42.16)	178 (43.82)	113 (37.73)	54 (33.78)		
**Family income to poverty ratio**, ***N*** **(%)**							
≤ 1.30	772 (27.33)	219 (31.72)	285 (25.79)	167 (23.33)	101 (29.79)	16.51	0.06
1.31–3.50	696 (46.39)	183 (42.96)	253 (43.52)	178 (54.20)	83 (47.05)		
> 3.50	194 (20.63)	57 (18.10)	76 (26.21)	36 (15.24)	25 (20.47)		
Missing	147 (5.66)	33 (7.23)	55 (4.48)	38 (7.23)	21 (2.68)		
**Residence**, ***N*** **(%)**							
Urban residence	835 (46.18)	240 (52.70)	302 (44.66)	205 (47.56)	88 (34.10)	6.25	0.10
Rural residence	974 (53.82)	252 (47.30)	367 (55.34)	214 (52.45)	141 (65.90)		
**Physical activity**^**b**^, ***N*** **(%)**							
Yes	970 (58.09)	232 (52.92)	368 (61.53)	237 (59.02)	133 (57.72)	3.61	0.31
No	839 (41.91)	260 (47.08)	301 (38.47)	182 (40.98)	96 (42.28)		
**BMI**^**c**^**, kg/m**^**2**^, ***N*** **(%)**							
Normal or below	1,209 (70.49)	330 (70.77)	448 (69.67)	276 (69.63)	155 (73.80)	1.31	0.97
Overweight	333 (18.34)	92 (17.68)	117 (18.41)	77 (19.01)	47 (18.27)		
Obesity	267 (11.17)	70 (11.55)	104 (11.93)	66 (11.37)	27 (7.93)		
**Carbohydrate intake, %kcal**, ***N*** **(%)**							
<45	392 (17.24)	120 (21.13)	152 (16.67)	78 (15.49)	42 (13.86)	4.03	0.67
45–65	1,252 (70.30)	325 (65.72)	459 (72.19)	298 (73.03)	170 (69.62)		
>65	165 (12.46)	47 (13.15)	58 (11.14)	43 (11.48)	17 (16.52)		
**WRAT-R**							
Arithmetic, *N* (%)							
<5% percentile scores	107 (4.13)	37 (6.00)	33 (3.32)	24 (2.16)	13 (6.10)	4.07	0.25
≥5% percentile scores	1,702 (95.87)	455 (94.00)	636 (96.68)	395 (97.84)	216 (93.90)		
Reading, *N* (%)							
<5% percentile scores	122 (3.89)	37 (5.35)	40 (3.18)	27 (2.10)	18 (6.16)	5.00	0.17
≥5% percentile scores	1,687 (96.11)	455 (94.65)	629 (96.82)	392 (97.90)	211 (93.84)		
**WISC-R**							
Block design*, N* (%)							
<5% percentile scores	94 (3.18)	31 (3.91)	22 (1.88)	19 (2.52)	22 (6.47)	6.02	0.11
≥5% percentile scores	1,715 (96.82)	461 (96.09)	647 (98.12)	400 (97.48)	207 (93.53)		
Digit span, *N* (%)							
<5% percentile scores	94 (3.30)	35 (5.45)	28 (2.30)	18 (1.18)	13 (5.50)	8.35	0.04
≥5% percentile scores	1,715 (96.70)	457 (94.55)	641 (97.79)	401 (98.82)	216 (94.50)		

Data source: NHANES III, National Health Interview Survey III, 1988-1994.

a All means and SEs for continuous variables and percentages and SEs for categorical variables were weighted, with the exception of the number of participants. Since all numbers were rounded, percentages may not total 100%. Data are presented as weighted means and standard errors in parentheses for continuous variables and frequencies and weighted percentages in parentheses for categorical variables. BMI, body mass index; SE, standard error.

b The adolescents were classified as being physically active most days of the week if adolescents reported participating in physical activity at least five times per week.

c Overweight: 85^th^ to less than the 95^th^ percentile; obesity: equal to or greater than the 95^th^ percentile.

The odds ratios (ORs) of SSB consumption with cognitive function in adolescents are listed in Table 2. In the crude model, compared with adolescents who drank SSBs 0–1 times per week, those who drank 4–7 times per week had better scores in arithmetic, reading, and digit span tests, with OR values of 0.35 (95% CI = 0.14–0.86), 0.38 (95% CI = 0.20–0.71), and 0.21 (95% CI = 0.09, 0.49), respectively. In the final model (model 3) additionally adjusted for age, sex, race/ethnicity, family reference person education years and marital status, poverty-income ratio, residence, physical activity, BMI, and carbohydrate intake, the ORs and 95% CIs (arithmetic, reading, and digit span tests) were not substantially changed. The ORs of SSB consumption and block design scores were invalid, but the risk of abnormal block design scores increased with the frequency of SSB intake after adjustment for three models (the *P*-values for trend were 0.049, 0.04, and 0.02, respectively).

**Table 2:** Association of intake of SSBs with cognitive tests in adolescents aged 12–16 years in the United States: NHANES III, 1988–1994.

**Scaled score**	**Frequency of intake of SSBs, OR (95%CI)**				
	**[0, 1)** **times/week**	**[1, 4)** **times/week**	**[4, 7) times/week**	**[7, 182]** **times/week**	***P* for trend**
**Arithmetic**					
Cases/Total	37/492	33/669	24/419	13/229	
Crude model	Reference	0.54	0.35	1.02	0.47
		(0.30–0.95)^*^	(0.14–0.86)^*^	(0.29–3.55)	
Model 1	Reference	0.53	0.33	0.97	0.45
		(0.31–0.90)^*^	(0.15–0.76)^*^	(0.34–2.79)	
Model 2	Reference	0.58	0.34	1.02	0.42
		(0.30–1.11)	(0.14–0.80) ^*^	(0.35–2.99)	
Model 3	Reference	0.59	0.36	1.05	0.41
		(0.31–1.10)	(0.16–0.82)^*^	(0.37–2.97)	
**Reading**					
Cases/Total	37/492	40/669	27/419	18/229	
Crude model	Reference	0.58	0.38	1.16	0.27
		(0.26–1.28)	(0.20–0.71) ^*^	(0.42–3.24)	
Model 1	Reference	0.57	0.36	0.99	0.43
		(0.26–1.25)	(0.20–0.67) ^*^	(0.33–2.93)	
Model 2	Reference	0.63	0.34	1.21	0.27
		(0.30–1.32)	(0.17–0.69) ^*^	(0.39–3.80)	
Model 3	Reference	0.64	0.35	1.22	0.26
		(0.33–1.24)	(0.18–0.70) ^*^	(0.42–3.60)	
**Block design**					
Cases/Total	31/492	22/669	19/419	22/229	
Crude model	Reference	0.47	0.64	1.70	0.05
		(0.19–1.18)	(0.25–1.60)	(0.56–5.21)	
Model 1	Reference	0.55	0.82	2.08	0.05^*^
		(0.22–1.36)	(0.31–2.12)	(0.62–7.04)	
Model 2	Reference	0.62	0.90	2.45	0.04^*^
		(0.25–1.56)	(0.33–2.47)	(0.66–9.04)	
Model 3	Reference	0.66	0.96	2.82	0.02^*^
		(0.27–1.61)	(0.38–2.41)	(0.82–9.71)	
**Digit span**					
Cases/Total	35/492	28/669	18/419	13/229	
Crude model	Reference	0.41	0.21	1.01	0.17
		(0.16–1.04)	(0.09–0.49)^*^	(0.44–2.31)	
Model 1	Reference	0.39	0.19	0.88	0.30
		(0.16–0.94)^*^	(0.08–0.44) ^*^	(0.36–2.15)	
Model 2	Reference	0.40	0.20	0.85	0.28
		(0.16–1.02)	(0.09–0.46)^*^	(0.37–2.00)	
Model 3	Reference	0.40	0.19	0.82	0.32
		(0.16–1.01)	(0.08–0.44)^*^	(0.34–1.98)	

* P < 0.05.

Model 1: adjusted for age and sex.

Model 2: Model 1 plus race/ethnicity, education level and marital status of family reference person, poverty–income ratio, residence, and physical activity.

Model 3: Model 2 plus body mass index and carbohydrate intake.

Stratified analyses showed that compared with normal or below BMI, among overweight or obese individuals, the frequency of SSB intake was a risk factor for abnormal digit span scores [(OR = 4.76, 95% CI = 1.19–18.96) vs. (OR = 0.35, 95% CI = 0.10, 1.25); *P* for interaction = 0.01]. The association did not significantly differ by sex or ethnicity (Table 3).

**Table 3:** Stratified analysis of the association of intake of SSBs with cognitive tests in adolescents aged 12–16 years in the United States: NHANES III, 1988–1994.

	**Frequency of intake of SSBs, OR (95%CI)**					
**Scaled score**	**[0, 1)** **times/week**	**[1, 4)** **times/week**	**[4, 7)** **times/week**	**[7, 182]** **times/week**	***P* for trend**	***P* for Interaction**
**Arithmetic**						
Cases/Total	37/492	33/669	24/419	13/229		
**Sex**						
Male	Reference	1.89	0.60	2.91	0.23	0.11
		(0.57–6.22)	(0.21–1.75)	(0.67–12.67)		
Female	Reference	0.24	0.28	0.38	0.58	
		(0.06–0.94)^*^	(0.07–1.12)	(0.08–1.73)		
**Race/ethnicity**						
Non-Hispanic white/Other	Reference	0.66	0.17	1.25	0.30	0.96
		(0.22–1.96)	(0.03–0.89)^*^	(0.31–4.97)		
Non-Hispanic black	Reference	0.34	0.72	0.42	0.27	
		(0.12–0.95)^*^	(0.35–1.48)	(0.18–0.98)^*^		
Mexican-American	Reference	1.23	0.97	0.65	0.48	
		(0.58–2.61)	(0.23–4.05)	(0.10–4.28)		
**BMI**						
Normal or below	Reference	0.69	0.25	1.18	0.31	0.59
		(0.27–1.77)	(0.09–0.71) ^*^	(0.42–3.35)		
Overweight or obesity	Reference	0.34	0.44	0.79	0.77	
		(0.09–1.30)	(0.12–1.67)	(0.10–6.43)		
**Reading**						
Cases/Total	37/492	40/669	27/419	18/229		
**Sex**						
Male	Reference	0.88	0.63	1.71	0.28	0.88
		(0.33–2.30)	(0.18–2.20)	(0.32–9.26)		
Female	Reference	0.48	0.16	1.33	0.30	
		(0.17–1.31)	(0.04–0.62) ^*^	(0.29–6.25)		
**Race/ethnicity**						
Non-Hispanic white/Other	Reference	1.10	0.10	0.96	0.64	0.97
		(0.38–3.17)	(0.02–0.58)	(0.21–4.37)		
Non-Hispanic black	Reference	0.43	0.98	1.32	0.21	
		(0.11–1.64)	(0.37–2.60)	(0.40–4.36)		
Mexican-American	Reference	0.63	0.66	0.72	0.92	
		(0.29–1.35)	(0.24–1.81)	(0.18–2.84)		
**BMI**						
Normal or below	Reference	0.84	0.42	1.80	0.17	0.27
		(0.36–1.92)	(0.18–0.98) ^*^	(0.51–6.38)		
Overweight or obesity	Reference	0.23	0.17	1.01	0.47	
		(0.08–0.66) ^*^	(0.03–0.80) ^*^	(0.14–7.17)		
**Block design**						
Cases/Total	31/492	22/669	19/419	22/229		
**Sex**						
Male	Reference	1.34	0.96	4.41	0.04^*^	0.97
		(0.21–8.68)	(0.22–4.19)	(0.72–27.03)		
Female	Reference	0.54	1.00	2.98	0.05	
		(0.20–1.44)	(0.36–2.79)	(0.66–13.45)		
**Race/ethnicity**						
Non-Hispanic white/Other	Reference	0.80	1.56	5.98	0.02^*^	0.65
		(0.18–3.57)	(0.33–7.28)	(0.81–44.08)		
Non-Hispanic black	Reference	0.47	0.32	0.91	0.22	
		(0.17–1.27)	(0.11–0.94)^*^	(0.39–2.12)		
Mexican-American	Reference	0.85	1.25	1.38	0.53	
		(0.24–3.04)	(0.20–7.88)	(0.23–8.30)		
**BMI**						
Normal or below	Reference	0.67	0.69	2.37	0.16	0.89
		(0.22–2.03)	(0.16–2.97)	(0.38–14.68)		
Overweight or obesity	Reference	0.48	1.11	4.47	0.02^*^	
		(0.11–2.02)	(0.24–5.12)	(0.67–29.86)		
**Digit span**						
Cases/Total	35/492	28/669	18/419	13/229		
**Sex**						
Male	Reference	0.47	0.23	1.02	0.20	0.66
		(0.16–1.41)	(0.07–0.71) ^*^	(0.30–3.45)		
Female	Reference	0.34	0.13	1.01	0.48	
		(0.11–1.04)	(0.04–0.45) ^*^	(0.23–4.33)		
**Race/ethnicity**						
Non-Hispanic white/Other	Reference	0.33	–^**^	0.78	0.28	0.20
		(0.08–1.46)		(0.21–2.87)		
Non-Hispanic black	Reference	0.57	0.35	0.22	0.25	
		(0.21–1.51)	(0.10–1.24)	(0.03–1.67)		
Mexican-American	Reference	0.48	1.40	0.69	0.87	
		(0.17–1.37)	(0.61–3.25)	(0.25–1.88)		
**BMI**						
Normal or below	Reference	0.37	0.09	0.35	0.74	0.01^*^
		(0.14–1.04)	(0.03–0.32) ^*^	(0.10–1.25)		
Overweight or obesity	Reference	0.24	0.52	4.76	0.01^*^	
		(0.07–0.85)^*^	(0.22–1.23)	(1.19–18.96)^*^		

* P < 0.05.

All sample sizes have been adjusted for weight. Stratified variables were not included in the model.

** The case of anomalous digit span scores is 0.

## Discussion

To the best of our knowledge, this is the first study examining the association of SSB intake with the subset score of WISC-R and WRAT. Based on a nationally representative population sample of the United States, we observed no significant association between the frequency of SSB intake and the scores of arithmetic, reading, and digit span at the overall level in adolescents. However, consuming SSBs 4–7 times per week was positively associated with arithmetic, reading, and digit span scores, with increasing frequency of SSB consumption, the risk of anomalous block design scores increased (*P* for trend = 0.02), and even after full adjustment, the association remained significant.

Although our findings were not in line with the hypothesis, these findings had some support from existing research. First, SSB consumption was found to be associated with improved cognitive function in the low and moderate intake groups but was negatively associated with higher intake levels. The Dietary Guidelines Advisory Committee in the United States have published a review in 2020 highlighting the harmful effects of high-level SSBs, by 2020, but the effect of low or moderate levels of SSB intake on health is yet to be determined (40). It has also been reported numerous times that there was no association between SSB consumption and cognitive function in children and adolescents (23, 24, 41, 42). For instance, the zero impact of sugar intake has been reported in lab-based laboratory studies of “sugar-responsive” children (41). Besides, a review by Benton concluded that there was no evidence of any negative effects of sugar on behavior (42).

The positive associations between lower consumption of SSBs and cognitive function observed in this study may be attributed to brain energy metabolism. The brain is metabolically demanding, and the weight of it only counts for 2% of total body weight but requires 20% of total energy intake due to its complex structure and processing needs (43). The preferred source of energy in the brain is glucose (44). The dynamic utilization of glucose by the cerebral cortex over the course of development suggests that the relative apportionment of glucose must also be dynamic (45), and since the glucose cannot be stored in the brain, at the right level of sugar intake, SSBs may raise the blood sugar level in the brain and boost brain metabolism, leading to higher cognitive scores (46). The result of a systematic review and meta-analysis of interventional studies also revealed modest beneficial effects of glucose on cognition, particularly recognition memory and attention processes (47).

Point estimates showed a negative relationship in the high SSB intake groups, although the ORs did not reach a statistically significant level. This suggests that excessive consumption of SSBs may damage cognitive function, and most studies have pointed out that excessive consumption of SSBs was related to neurological decline (21, 22, 48). A study has reported that consumption of sugary beverages in early childhood is negatively associated with KBIT-II language scores in middle childhood [−2.4 points per serving/day, 95% CI (−4.3, −0.5)] (48). Notably, in our study, it was found that block design scores reflecting short-term memory and attentional function decreased with increasing consumption of SSBs, showing a significant dose-response relationship, which may be related to SSBs affecting memory-related brain regions. Studies in animal models have shown that deleterious effects of long-term sugar intake on memory deficits and hippocampal neurogenesis (49), 2 months of an HFS (high-fat, refined sugar) diet, were sufficient to reduce hippocampal levels of BDNF and spatial learning performance (50), and excessive consumption of added sugars, especially HFCS-55, during the adolescent period of development, negatively affect hippocampal function, metabolic outcomes, and neuroinflammation (19). In addition, Jacobson et al. found an independent association between cognitive changes and time-weighted HbA_1c_ in diabetic people, which they believed may reflect the deleterious effects of high brain glucose levels on neuronal integrity (51). Glucose metabolism increased the glutamate-glutamine cycle (52); therefore, higher cerebral blood glucose may lead to increased prefrontal Glx (an excitatory neurotransmitter that causes neuronal damage at high concentrations) concentrations (53), which was associated with reduced cognitive performance (54). This might explain the increased risk of abnormal block design scores with consumption of SSBs as observed in our study.

The relationship between sugar intake and cognitive function in adolescents is not well understood to date. Existing literature had inconsistent and unclear cutoff values for SSB consumption and failed to reveal the true association between SSBs and cognitive function, and this should be addressed in further studies along with investigation regarding the potential underlying mechanism.

This study has some strengths. To the best of our knowledge, this is the first report of an association of SSB intake with cognitive function in adolescents. In addition, the NHAENS is a nationally representative survey that provides reliable data to explore this association. There are also several limitations to be noted. First, this is a cross-sectional observational study, and we cannot infer the causal relationship between SSB consumption and cognitive function of adolescents. Second, the consumption of SSBs was assessed on a frequency basis, making it impossible to determine specific intake volume, which may affect the correlation of outcomes. Finally, the use of self-reported FFQ to obtain dietary intake data may be subject to recall bias, thus introducing errors into our estimation model.

## Conclusion

In this study, no association was found between the frequency of consumption of SSBs and arithmetic, reading, and digit span at the overall level in adolescents. The positive associations of SSBs at moderate level intake with better scores in arithmetic, reading, and digit span, but with increasing frequency of SSB consumption, the risk of anomalous block design scores increased among US adolescents. Further investigation is warranted to confirm the association and mechanism between SSBs and cognitive function among adolescents.

## Data availability statement

The datasets presented in this study can be found in online repositories. The names of the repository/repositories and accession number(s) can be found at: https://www.cdc.gov/nchs/nhanes/index.htm.

## Ethics statement

The studies involving human participants were reviewed and approved by the National Center for Health Statistics Research Ethics Review Board. Written informed consent to participate in this study was provided by the participants' legal guardian/next of kin.

## Author contributions

WY has full access to all the data in this study and assumes responsibility for study supervision. WY and QC conceptualized and designed the study, collected and analyzed data, carried out the initial analyses, and reviewed and revised the manuscript. XY, YX, and JZ acquired, analyzed, interpreted the data, and drafted the initial manuscript. All authors critically revised manuscripts of significant intellectual content. All authors approved the final manuscript as submitted and agreed to be accountable for all aspects of the work.

## Conflict of interest

The authors declare that the research was conducted in the absence of any commercial or financial relationships that could be construed as a potential conflict of interest.

## Publisher's note

All claims expressed in this article are solely those of the authors and do not necessarily represent those of their affiliated organizations, or those of the publisher, the editors and the reviewers. Any product that may be evaluated in this article, or claim that may be made by its manufacturer, is not guaranteed or endorsed by the publisher.
